# Cell Migration According to Shape of Graphene Oxide Micropatterns

**DOI:** 10.3390/mi7100186

**Published:** 2016-10-14

**Authors:** Sung Eun Kim, Min Sung Kim, Yong Cheol Shin, Seong Un Eom, Jong Ho Lee, Dong-Myeong Shin, Suck Won Hong, Bongju Kim, Jong-Chul Park, Bo Sung Shin, Dohyung Lim, Dong-Wook Han

**Affiliations:** 1 Department of Cogno-Mechatronics Engineering, College of Nanoscience and Nanotechnology, Pusan National University, Busan 46241, Korea; 01048470363@naver.com (S.E.K.); choel15@naver.com (Y.C.S.); sueom89@gmail.com (S.U.E.); pignunssob@naver.com (J.H.L.); swhong@pusan.ac.kr (S.W.H.); bosung@pusan.ac.kr (B.S.S.); 2 Cellbiocontrol Laboratory, Department of Medical Engineering, Yonsei University College of Medicine, Seoul 03722, Korea; kimminsec@nate.com (M.S.K.); parkjc@yuhs.ac (J.-C.P.); 3 Research Center for Energy Convergence Technology, Pusan National University, Busan 46241, Korea; dmshin@pusan.ac.kr; 4 Dental Life Science Research Institute, Seoul National University Dental Hospital, Seoul 03080, Korea; bjkim016@gmail.com; 5 Department of Mechanical Engineering, Sejong University, Seoul 05006, Korea

**Keywords:** photolithography, meniscus-dragging deposition, graphene oxide, micropatterns, cell migration

## Abstract

Photolithography is a unique process that can effectively manufacture micro/nano-sized patterns on various substrates. On the other hand, the meniscus-dragging deposition (MDD) process can produce a uniform surface of the substrate. Graphene oxide (GO) is the oxidized form of graphene that has high hydrophilicity and protein absorption. It is widely used in biomedical fields such as drug delivery, regenerative medicine, and tissue engineering. Herein, we fabricated uniform GO micropatterns via MDD and photolithography. The physicochemical properties of the GO micropatterns were characterized by atomic force microscopy (AFM), scanning electron microscopy (SEM), and Raman spectroscopy. Furthermore, cell migration on the GO micropatterns was investigated, and the difference in cell migration on triangle and square GO micropatterns was examined for their effects on cell migration. Our results demonstrated that the GO micropatterns with a desired shape can be finely fabricated via MDD and photolithography. Moreover, it was revealed that the shape of GO micropatterns plays a crucial role in cell migration distance, speed, and directionality. Therefore, our findings suggest that the GO micropatterns can serve as a promising biofunctional platform and cell-guiding substrate for applications to bioelectric devices, cell-on-a-chip, and tissue engineering scaffolds.

## 1. Introduction

Photolithography is a unique process that can facilitate the manufacturing of micro-sized patterns on various substrates. This process has significantly higher resolution than other patterning methods as well as good reproducibility and efficiency from an economical and temporal perspective. Therefore, photolithography has been widely used as a patterning process to fabricate semi-conductors, stretchable devices, and medical devices [1,2].

Graphene oxide (GO), the oxidized form of graphene, is a carbon-based hexagonal structure with oxygen containing groups such as carboxyl, hydroxyl, and epoxy groups [3,4,5]. GO has good dispersion in aqueous solutions, which is useful for uniformly coating GO [6]. In addition, GO presents an open surface for noncovalent interactions with biomolecules. Recent research has shown that GO can enhance cellular behaviors including attachment, proliferation, and differentiation due to various functional groups on its surface that can promote cellular behaviors through interactions with cells [7,8,9,10]. Based on this reason, it is inferred that GO-coated substrates can induce aligned array of cells. GO-coated substrates can be fabricated by various coating techniques, including filtration/transfer-based film formation, spin coating, air-spraying, dip coating, Langmuir–Blodgett deposition, and wire-wound rod coating, for electrical devices and medical applications. Some of these methods produce relatively non-uniform thin films because of the aggregation of GO particles. In addition, the majority of these techniques for the production of GO is not easy to scale over a large area. Recently, the meniscus-dragging deposition (MDD) technique, which is a microliter-scale solution process for fabricating thin film-coated substrates with a significant decrease of the solution consumption, has been spotlighted because the process can easily and uniformly fabricate GO-coated substrates. The MDD technique can develop highly uniform GO films on substrates by dragging the meniscus of a GO suspension trapped between a deposition plate and a coating substrate in an alternating back-and-forth motion [11,12,13,14]. Therefore, in this study, we fabricated GO micropatterns on a glass substrate via MDD and photolithography techniques and investigated their effects on cell migration.

Directional cell migration is critical for many important biological processes, including angiogenesis, tumor metastasis, wound healing, and nerve regeneration. Most work on the directional control of cell motility has focused on the role of gradients of motility factors such as platelet-derived growth factor, fibroblast growth factor, and epidermal growth factor, with the general concept that cells physically move up the gradient of a soluble attractant. These factors promote cell migration by activating members of the Rho family of GTPases-Rac and CDC43, which induce the formation of actin-based lamellipodia, filopodia, and fascin-containing microspikes that drive cell extension. On the other hand, recently, many studies have been concerned on the development of micropatterns that can induce cell migration to a desired direction. The specific micropatterns can induce and guide the cell migration by providing physical and topographical cues. In addition, the micropatterned substrates are effectively and consistently able to provide guidance cues [15,16,17]. Therefore, we speculated that the GO micropatterns might guide the cell migration [18,19,20,21].

Herein, we fabricated the GO micropatterns on a slide glass evenly using MDD and photolithography techniques. In addition, L-929 fibroblasts were cultured on the GO micropatterns to explore the effects of the GO micropatterns on cell migration. Furthermore, the difference in cell migration according to the pattern shape was investigated to explore the potential of GO micropatterns as a biofunctional platform for bioelectric devices and tissue engineering applications.

## 2. Materials and Methods

### 2.1. Preparation of GO-Coated Substrates and GO Micropatterns

A GO solution was purchased from Sigma-Aldrich Co. (St. Louis, MO, USA). To prevent the defects of GO during photolithography, 25 mm × 75 mm slide glass was pre-treated by placing it into a piranha solution (H_2_SO_4_:H_2_O_2_ = 3:1) for 30 min. The deposition plate was placed on the coating substrate at an angle of 30°. The 120-μL GO solution (4 mg/mL in distilled water) was injected into the wedge between the plate and slide glass. The deposition plate was moved linearly in a back-and-forth motion at a constant speed of 15 mm/s in a 35% humidified atmosphere to deposit the GO on the substrate. After the coating process, GO-coated slide glass was dried in a vacuum oven at 80 °C for 30 min.

Positive photoresists (PRs, az5214e) were spin-coated on the GO-coated slide glass and soft baked at 95 °C for 5 min. Next, the substrates were exposed to 20 mW of ultra-violet (UV) lights for 6 s through a micropatterned chrome mask. During the developing step, the exposed PRs were dissolved by a developer (AZ 300 MIF, AZ Electronic Materials, Branchburg, NJ, USA). Then, 100 sccm of O_2_ plasma was applied to the remaining GO between the PRs and the slide glass for 6 min. After all of these steps, the substrates were washed with acetone and dried under N_2_ gas to remove the PRs.

### 2.2. Physicochemical Characterization of GO Micropatterns

The topography of the GO-coated slide glass using the MDD method was characterized by atomic force microscopy (AFM, NX10, Park Systems Co., Suwon, Korea) in air at room temperature (RT). Imaging was performed in non-contact mode with a Multi 75 silicon scanning probe at a resonant frequency of ~300 kHz. Image analysis was performed using XEI Software (version 1.7.1, Park Systems Co.). To examine the morphology of GO micropatterns, the GO micropatterns on substrates were observed with a field emission scanning electron microscope (FESEM, Hitachi S-4700, Tokyo, Japan) at an accelerating voltage of 5 kV. Compositional analysis of the GO micropatterns was performed via Raman spectroscopy (Micro Raman PL Mapping System, Dongwoo Optron Co., Ltd., Kwangju-si, Korea) with excitation at 532 nm using an Ar-ion laser with a radiant power of 5 mW at RT.

### 2.3. Time-Lapse Imaging and Analysis of Cell Migration on GO Micropatterns

L-929 fibroblasts were purchased from the American Type Culture Collection (ATCC, Rockville, MD, USA) and routinely maintained in Dulbecco’s modified Eagle’s medium (Welgene, Daegu, Korea) supplemented with 10% fetal bovine serum (Welgene) and 1% antibiotic–antimycotic solution (including 10,000 units of penicillin, 10 mg of streptomycin, and 25 μg of amphotericin B per mL, Sigma-Aldrich Co.) at 37 °C in a humidified atmosphere containing 5% CO_2_.

Cell migration images were captured with an Olympus IX81 inverted fluorescence microscope (Olympus Optical Co., Osaka, Japan). Captured images were imported into ImageJ (ImageJ, version 1.37 by Wayne Rasband, National Institutes of Health, Bethesda, MD, USA), and image analysis was carried out with the manual tracking and chemotaxis tool plug-in (version 1.01, distributed by ibidi GmbH, Munchen, Germany) [22,23,24,25]. The *XY* coordinates of each cell were obtained using the manual tracking plug-in in the ImageJ program. Only one cell of each group was tracked and the center of each cell was tracked 3 times to obtain accuracy. The tracked data were imported into the chemotaxis plug-in. The cell migration speed was computed automatically, and the cell migration pathway was plotted with the chemotaxis tool. The migration speed indicates how fast cells move in response to the stimulation, calculated using the total length of the migration path divided by the total observation time. The distance is the total length of the cell migration path during the observation time. Cells undergoing division, death, or migration outside the field of view were excluded from the analysis.

### 2.4. Statistical Analysis

All variables were tested in three independent cultures for each in vitro experiment, which was repeated twice (*n* = 6). The quantitative data are given as the mean ± standard deviation (SD). A one-way analysis of variance (ANOVA, SAS Institute Inc., Cary, NC, USA) was performed to analyze the difference in cell migration according to the pattern shape by a Tukey’s honestly significant difference (HSD) test. A value of *p* < 0.05 was considered statistically significant.

## 3. Results and Discussion

### 3.1. Preparation of GO-Coated Substrates and GO Micropatterns

**Figure 1:**
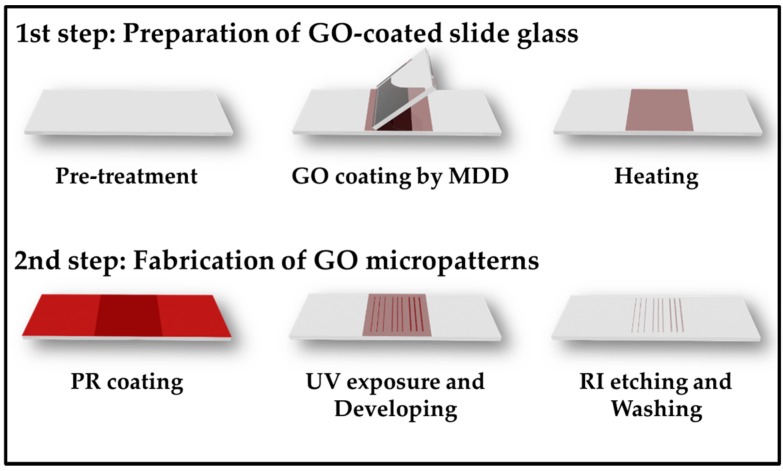
Schematic illustration for the preparation of graphene oxide (GO)-coated slide glass and GO micropatterns. PR: positive photoresists; MDD: meniscus-dragging deposition; UV: ultra-violet.

**Figure 2:**
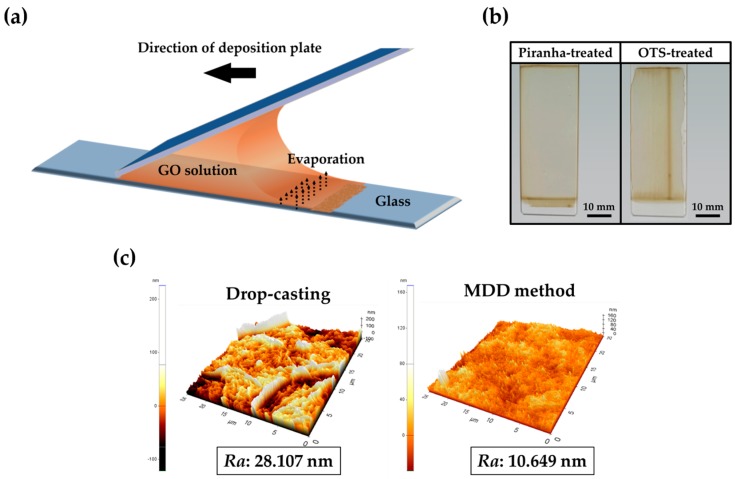
Preparation of GO-coated slide glass using the MDD method. (**a**) Schematic illustration of MDD technique; (**b**) Digital photographs of GO-coated slide glass in different pre-treatments; (**c**) Atomic force microscope (AFM) images and surface roughness (*Ra*) of GO-coated slide glass according to GO coating methods. OTS: octadecyltrichlorosilane.

The procedures of fabrication of GO micropatterns on a slide glass are divided into two main steps (Figure 1). First, to produce uniform GO layers on the substrate, the MDD method was used. Figure 2a displays a schematic illustration of the MDD process, which can allow GO particles to be uniformly coated on the substrate. In brief, the deposition plate was moved at a constant angle and speed to deposit the GO solution on the substrate. Then, there were differences in evaporation ratio because of the meniscus phenomena, and the GO particles were evenly coated on the surface of substrate.

Figure 2b displays digital photographs of GO-coated slide glass with different pre-treatments. The piranha-treated slide glass was coated uniformly. However, when the slide glass was pre-treated with octadecyltrichlorosilane (OTS), GO was coated non-uniformly. This could be in part due to the hydrophobicity of OTS. This result confirmed that the hydrophilic surface of slide glass is more suitable for GO coating than a hydrophobic surface [26,27]. We chose the piranha-treated slide glass for the manufacturing of uniform GO micropatterns. Figure 2c displays the AFM images of GO-coated slide glass surface with different methods of GO coating. The surface of the GO-coated slide glass using the MDD method was formed uniformly on slide glass and had a lower average of surface roughness than the GO-coated slide glass using drop-casting. Lower roughness means that the GO was highly uniformly coated on the slide glass without an aggregation of GO particles. Therefore, it is indicated that the MDD method with proper conditions is a suitable procedure for manufacturing uniformly GO-coated slide glass.

The second step of photolithography is PR coating and the developing process (Figure 1). A PR is a photopolymer resin that can regulate the cross-linking between the molecules via light energy. After PR coating, the light source was passed through a chrome mask that carved specific micropatterns. Then, the cross-linked bonds between PRs became weak, and the PRs could be dissolved in the developer solution.

Finally, the substrate containing the GO layers and the micropatterned PR was exposed to O_2_ plasma to form GO micropatterns because the GO layers under the micropatterned PR did not react with the plasma due to the protection of the PR. After washing with acetone and drying under N_2_ gas, the uniform GO micropatterns were obtained.

### 3.2. Physicochemical Characeristics of GO Micropattenrs

**Figure 3:**
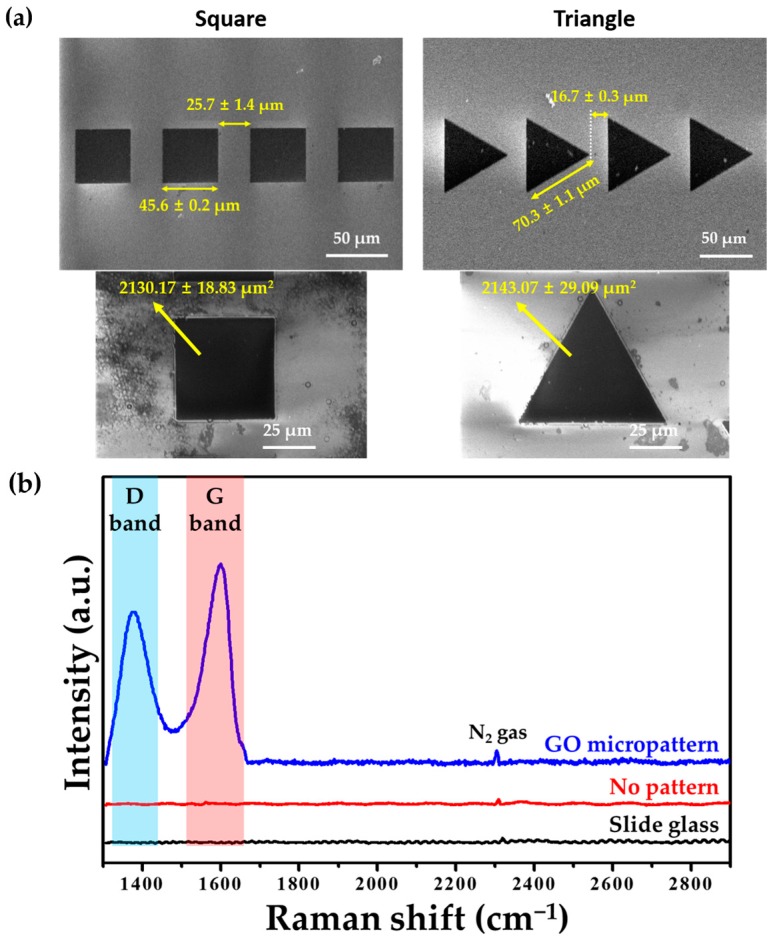
Physicochemical characteristics of GO micropatterns. (**a**) Field emission scanning electron microscopy (FESEM) images and (**b**) Raman spectra of GO micropatterns. Characteristic bands of GO including D and G bands were observed in GO micropatterns.

Figure 3a showed the surface morphologies from FESEM. In this study, two types of GO micropatterns—square and triangle shapes—were designed on slide glass. The gaps between the square and triangle patterns were 25.7 ± 1.4 μm and 16.7 ± 0.3 μm, respectively. They were close enough for extended lamellipodia to reach into adjacent micropatterns, but far enough to momentarily confine individual cells. The side length of the square pattern was shorter than that of the triangle pattern. Although side lengths of two type patterns are different, the area of GO micropatterns is highly similar because it is important that the same quantity of GO is coated on each pattern to investigate the effects of GO.

Figure 3b displays the Raman spectra of the GO micropatterns. It was demonstrated that the spectrum of the GO micropatterns included characteristic bands of GO: the D and G bands. These characteristic peaks of GO were not shifted in the GO micropatterns. The D and G bands were observed at approximately 1390 and 1600 cm^−1^, which were assigned to the vibration of sp^3^ carbon atoms and the structural defects of the sp^2^ carbon domains, respectively. In addition, in general, the intensity ratio of the D and G bands (*I*_D_/*I*_G_ value) of GO is less than 1 because the GO has many defects on its surface [28,29,30]. As shown in Figure 3b, the *I*_D_/*I*_G_ value of GO was less than 1, which is in accordance with previous studies. Therefore, it was demonstrated that the GO micropatterns were successfully formed on the slide glass.

### 3.3. Effects of GO Micropatterns on Cell Migration

**Figure 4:**
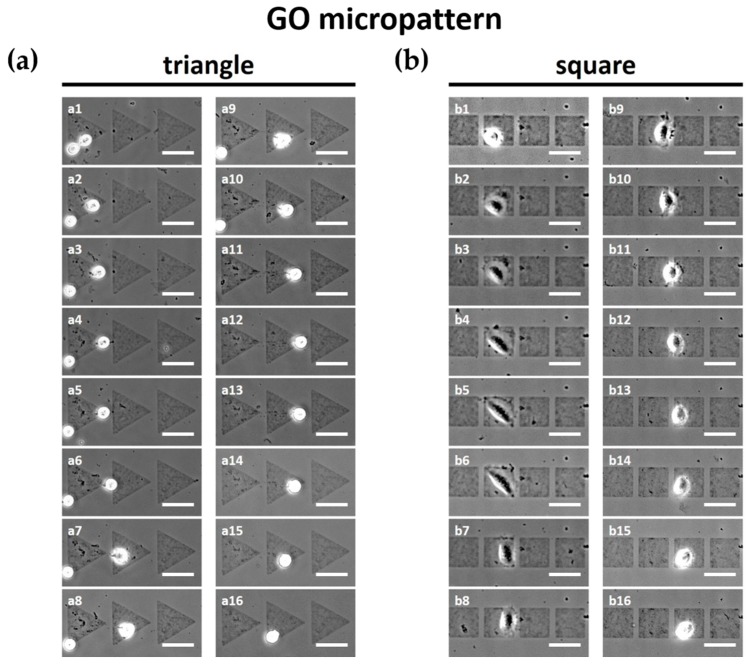
Time-lapse images of L-929 fibroblasts on (**a**) triangle and (**b**) square GO micropatterns for 12 h. Scale bars are 50 µm. (**a**) L-929 fibroblasts on triangle GO micropatterns moved from left to right initially (a1–a8); and then moved backwards to the left hand side (a9–a16). (**b**) The migration distance of L-929 fibroblasts on square GO micropatterns was significantly shorter than that on the triangle GO micropatterns (b1–b16).

The migration of L-929 fibroblasts on GO micropatterns was investigated via optical microscopy (Figure 4). We found that, firstly, L-929 fibroblasts moved on micropatterns rather than the unpatterned slide glass region due to the GO. This can be attributed the fact that the functional groups of the GO surface can provide a favorable environment for cell attachment and growth. In a previous study, it was found that GO can regulate cellular responses and attract the cells because GO has many hydrophilic functional groups including hydroxyl, carboxyl, and epoxy groups [3,4,7,9]. As a result, the interactions between GO and cells can be promoted via the hydrophilic functional groups of the GO surface, which results in successful cell adhesion on the GO micropatterns.

The cells on GO micropatterns gradually migrated following the GO micropatterns. As shown in Figure 4a, the L-929 fibroblasts on the triangle micropatterns migrated from left to right initially, and then moved backwards to the left hand side. In addition, interestingly, the cells moved along an oblique side of the triangle micropatterns, toward the vertex of the triangle patterns continuously. It has been revealed that the asymmetric relative positioning of the micropatterns can provide both path and directionality. A previous study has demonstrated that lamellipodia attachment has an influence on the shape of substrate [31,32]. It was found that the migration distance of the cells on the symmetric square micropatterns was significantly (*p* < 0.05) shorter than that on the triangle micropatterns (Figure 4b), although the cells on the square micropatterns also migrated to the next micropattern (Videos S1 and S2).

**Figure 5:**
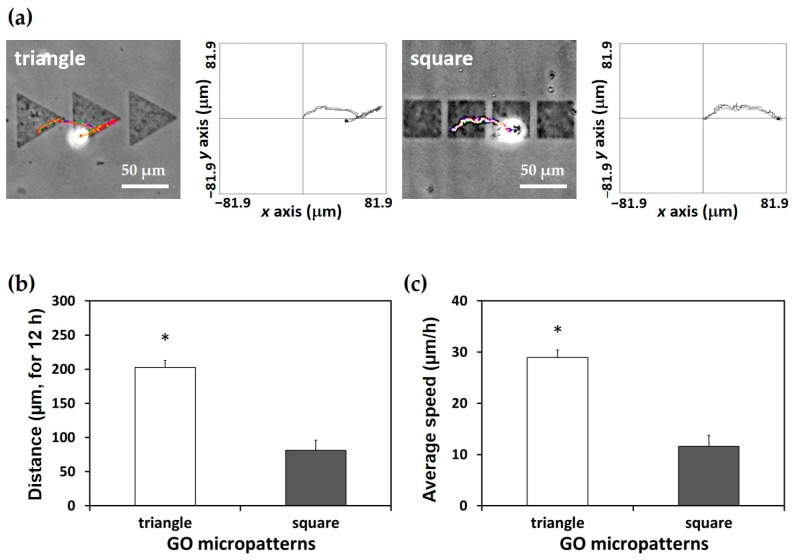
Quantitative analysis of cell migration. (**a**) Trajectories of L-929 fibroblasts on triangle and square GO micropatterns. (**b**) Migration distance and (**c**) average migration speed of L-929 fibroblasts on GO micropatterns. An asterisk (*) denotes a significant difference compared to the square GO micropatterns (*p* < 0.05).

To quantitatively analyze the migration of the L-929 fibroblasts on the GO micropatterns, trajectories, migration distance, and average migration speed were calculated. Figure 5a presents the trajectories of the cells on each micropattern for 12 h. It is demonstrated that cells on the triangle micropatterns tended to move along an oblique side of the triangle GO micropatterns. This can be partly explained by the fact that the lamellipodia of cells tends to reach the edge of the shape or the sharp part [33,34]. Furthermore, as shown in Figure 5b,c, both migration distance and speed of the cells on the triangle micropatterns were significantly (*p* < 0.05) higher than those on the square micropatterns. In addition, it is related to a change of cell morphology depending on the topography of the patterns [31,32]. In Figure 4, the cell morphologies on the triangle and square patterns are apparently different. The cell morphology on the square patterns was more spread on the GO micropatterns than that on the triangle patterns. This could also affect the slower speed and shorter distance of the cell migration on the square patterns.

Taken together, our results demonstrated that the cell migration was strongly dependent on the shape of the GO micropatterns, and the triangle GO micropatterns were more suitable for enhancing cell migration in terms of migration distance, speed, and directionality. Consequently, it is suggested that the GO micropatterns with specific geometrical cues can consistently regulate and guide cell migration without any chemical factors.

## 4. Conclusions

The aim of the present study was to develop uniform GO micropatterns and to explore their effects on cell migration. The triangle and square GO micropatterns were finely fabricated using MDD and photolithography techniques. In addition, our findings revealed that the cell migration can be guided by the GO micropatterns having specific geometrical cues, and the triangle GO micropatterns can enhance the cell migration distance, speed, and directionality compared with the square GO micropatterns. Therefore, it is suggested that the GO micropatterns can be employed as a promising biofunctional platform and cell-guiding substrate for applications to bioelectric devices, cell-on-a-chip, and tissue engineering scaffolds.

## Supplementary Materials

The following are available online at www.mdpi.com/2072-666X/7/10/186/s1: Video S1: Time-lapse video of L-929 fibroblasts on triangle GO micropatterns for 12 h; Video S2: Time-lapse video of L-929 fibroblasts on square GO micropatterns for 12 h.
